# Younger age is associated with cardiovascular pathological phenotype of severe COVID-19 at autopsy

**DOI:** 10.3389/fmed.2023.1327415

**Published:** 2024-01-08

**Authors:** Fernando R. Giugni, Amaro N. Duarte-Neto, Luiz Fernando F. da Silva, Renata A. A. Monteiro, Thais Mauad, Paulo H. N. Saldiva, Marisa Dolhnikoff

**Affiliations:** ^1^Departamento de Patologia, Faculdade de Medicina, Universidade de São Paulo, São Paulo, Brazil; ^2^Division of Cardiology, University of Texas Southwestern Medical Center, Dallas, TX, United States; ^3^LIM 05 - Laboratório de Patologia Ambiental e Experimental, Faculdade de Medicina, Universidade de São Paulo, São Paulo, Brazil; ^4^SVOC - Serviço de Verificação de Óbitos da Capital, Universidade de São Paulo, São Paulo, Brazil

**Keywords:** children, MIS-C, myocarditis, pathology, SARS-CoV-2

## Abstract

**Introduction:**

COVID-19 affects patients of all ages. There are few autopsy studies focusing on the younger population. We assessed an autopsy cohort aiming to understand how age influences pathological outcomes in fatal COVID-19.

**Methods:**

This study included autopsied patients, aged 6 months to 83 years, with confirmed COVID-19 in 2020–2021. We collected tissue samples from deceased patients using a minimally invasive autopsy protocol and assessed pathological data following a systematic approach.

**Results:**

Eighty-six patients were included, with a median age of 55 years (IQR 32.3–66.0). We showed that age was significantly lower in patients with acute heart ischemia (*p* = 0.004), myocarditis (*p* = 0.03) and lung angiomatosis (*p* < 0.001), and significantly higher in patients with exudative diffuse alveolar damage (*p* = 0.02), proliferative diffuse alveolar damage (*p* < 0.001), lung squamous metaplasia (*p* = 0.003) and lung viral atypia (*p* = 0.03), compared to patients without those findings. We stratified patients by their age and showed that cardiovascular findings were more prevalent in children and young adults. We performed principal component analysis and cluster of pathological variables, and showed that cardiovascular variables clustered and covariated together, and separated from pulmonary variables.

**Conclusion:**

We showed that age modulates pathological outcomes in fatal COVID-19. Younger age is associated with cardiovascular abnormalities and older age with pulmonary findings.

## 1 Introduction

COVID-19 affects patients of all ages. Up to early September 2022, the COVID-19 pandemic had affected more than 500 million individuals. Brazil occupied the third place among countries with the highest number of cases (more than 30 million cases), following the USA and India; and the second position in number of deaths attributed to COVID-19, with more than 650 thousand deaths. In Brazil, as in the rest of the world, deaths attributed to COVID-19 occur mainly in people over 60 years of age (1). Morbi-mortality increases with age, especially among the elderly, as they have greater prevalence of comorbidities, more extensive pulmonary disease and higher levels of inflammatory biomarkers (2–4).

Children represent a small fraction of COVID-19 cases. They were no more than 2% of laboratory-confirmed patients in early pandemic, but have increased in proportion after wide-spread testing, representing 8.5–13.5% of total global cases (5). In general, children are either asymptomatic or present with mild symptoms, such as cough and fever (5, 6). Hospitalization is uncommon and ICU admission rates are very low (7). In addition to the small prevalence of comorbidities, other mechanisms may explain differences in severity and incidence of COVID-19 in children compared to adults, such as angiotensin-converting enzyme-2 (ACE2) expression, efficient innate immune response, trained immunity and less lymphoid depletion (5, 8). However, although far less frequent, children and young adults may also present with severe disease (9).

During the pandemic, autopsy studies have helped us understand the morphological bases of clinical manifestations in COVID-19. The main pathological findings in the lungs are different phases of diffuse alveolar damage (DAD) (acute, fibroproliferative and/or fibrosis) with significant epithelial cytopathic effects, and vascular damage, mainly represented by microthrombosis and capillary angiomatosis (10).

COVID-19 also frequently affects other systems, such as cardiovascular, renal, endocrine, neurological and gastrointestinal (11). Extrapulmonary abnormalities may be remarkable at autopsy, often secondary to previous diseases or to refractory hypoxemia, systemic inflammation and shock, which significantly contribute to the high morbidity of COVID-19 (11). Occasionally, extrapulmonary damage may be directly attributed to SARS-CoV-2 infection, with detection of viral RNA and antigens in tissues. For example, SARS-CoV-2-induced myocarditis and thrombi in the myocardial vascular bed have been frequently described among severe cases and are associated with higher mortality (12). Clinical presentations of COVID-19 with predominant cardiovascular manifestations have been defined as Acute COVID-19 Cardiovascular Syndrome (13, 14). In the particular presentation of multisystem inflammatory syndrome in children (MIS-C) and in adults (MIS-A), SARS-CoV-2 infection can induce an atypical clinical-pathological picture, in which the extrapulmonary damage is more prominent than that observed in the lungs, with myocarditis, pericarditis, encephalitis, gastrointestinal manifestations and mucocutaneous disease (15, 16). Yet, the pathology of visceral lesions in these COVID-19-associated syndromes is poorly described.

Most autopsy studies of COVID-19 include only adult patients, mostly elderly, with scarce data on pathological findings among children and adolescents with severe disease (17–19). We have previously analyzed the autopsy of five children that died of COVID-19, two of them with severe acute respiratory disease and DAD, and three patients with MIS-C with the involvement of several organs, including the heart, intestine and central nervous system (17). The presence of SARS-CoV-2 in several organs of those children, associated with cellular ultrastructural changes, suggested that a direct effect of SARS-CoV-2 on tissues may be involved in the pathogenesis of MIS-C.

No autopsy studies have evaluated the morphological presentation of fatal COVID-19 within a broad age spectrum. Since age may influence the clinical presentation of severe COVID-19, our hypothesis is that it affects the disease’s pathology as well. In this study, we aimed to understand how age influences pathological outcomes in fatal COVID-19. To do so, we evaluated a cohort of 86 patients who died of COVID-19, aged 6 months to 83 years, and assessed the associations of age with pulmonary and cardiovascular pathological findings.

## 2 Materials and methods

This study was approved by the HC-FMUSP Ethical Committee and Institutional Review Board (protocol number 3.951.904). Patients’ next-of-kin gave written consent for the autopsy procedure. The investigation conforms with the principles outlined in the *Declaration of Helsinki.*

This is an autopsy cohort study that started on March, 18th 2020, aiming to study the pathology of COVID-19 on deceased patients referred to the central morgue of the city of São Paulo – the Death Verification Service at the University of São Paulo.

### 2.1 Patients

From March to December 2020, 4,633 patients with confirmed COVID-19 were hospitalized in our institution, with 1,573 deaths (34% mortality rate). From these, we performed 80 autopsies that were requested by the HC-FMUSP clinical staff; all of them were included in the study, comprising patients with the classic presentation of respiratory failure, and also those with complications or different presentations of the disease, such as MIS-C and MIS-A. To increase our sample of younger patients, we also included all the six autopsies from patients with age < 40y who died in 2021 (total = 86 patients). The confirmed cases of COVID-19 were defined according to the World Health Organization: cases with molecular confirmation of SARS-CoV-2 infection, regardless of clinical signs, through RNA detection in *premortem* or *postmortem* nasopharyngeal/oropharyngeal swab, or postmortem sampled tissues by real-time reverse-transcription polymerase chain reaction (rRT-PCR) (20).

We reviewed patients’ medical records to retrieve information about their age, sex, past medical history, body mass index and interval from first symptoms to death. MIS-C and MIS-A were defined according to the Centers for Disease Control and Prevention (21, 22).

### 2.2 Autopsies and microscopic analysis

For safety reasons, during the COVID-19 pandemic in 2020 and 2021, we performed ultrasound-guided minimally invasive autopsies (MIA-US), and our protocol is described in details elsewhere (10, 23). All autopsies were conducted by physicians from the Pathology Department of the University of São Paulo, and the procedures were performed at the HC-FMUSP Research Center Imaging Platform in the Autopsy Room.^1^ All autopsies were performed within a post-mortem period of less than 24 h. We used portable SonoSite M-TurboR (Fujifilm, Bothell, Washington) US equipment with C60x (5–2 MHz Convex) multifrequency broadband transducers and generation of Digital Imaging and Communications in Medicine standard images. We collected tissue specimens from the lungs, heart, brain, kidneys, liver and spleen, and occasionally from other organs. They were fixed with formalin and embedded in paraffin. Fresh lung tissue samples were frozen immediately after collection to perform rRT-PCR for SARS-CoV-2 detection. Hematoxylin–eosin staining was routinely performed for all tissues; other stains were performed when necessary, mainly for identification of secondary infections. Immunohistochemistry, electron microscopy and tissue rRT-PCR were performed on selected cases to confirm the diagnosis of COVID-19 when it was not confirmed ante-mortem (23).

Histopathological examination was performed by a team of pathologists (ANDN, LFFS, TM, PHNS, and MD) with large experience in autopsy and pulmonary pathology, following a systematic approach for histopathological tissue assessment. We pre-specified pathological alterations for each organ to be classified as absent/present or as absent/moderate/severe. Criteria for classification of pulmonary and acute/subacute cardiac alterations were described in detail in previous published papers (10, 24), and can be summarized as follows: (a) exudative diffuse alveolar damage (DAD): interstitial and/or intra-alveolar edema, interstitial pneumonitis, alveolar hemorrhage, intra-alveolar fibrin deposition/hyaline membranes, and type II pneumocyte hyperplasia; (b) proliferative DAD: variable degree of fibroblastic proliferation within the interstitium and/or alveolar spaces, including loose aggregates of fibroblasts admixed with scattered inflammatory cells, collagen deposition, densely fibrotic areas, admixed with areas with hyaline membranes; (c) squamous metaplasia: squamous epithelial lining in alveolar spaces; (d) viral atypia: viral cytopathic effects in pneumocytes; (e) vascular thrombosis: thrombosis in small and medium pulmonary arteries; (f) “cystic” alterations: honeycomb pattern in pulmonary areas with interstitial fibrosis; (g) angiomatoid pattern: expanded alveolar septa due to capillary filled with fibrinous microthrombi (25); (h) heart acute ischemia: variable areas of cardiomyocytes with hyaline degeneration, advanced nuclear karyolysis, and interstitial edema (26); (i) myocarditis: interstitial myocardial infiltrate by mononuclear cells and/or polymorphonuclear cells; (j) heart thrombosis: thrombi in small and medium heart vessels (27).

### 2.3 Statistical analysis

We described data as frequencies for qualitative data and median/interquartile-range for quantitative data when the distribution was not normal.

To assess if age was associated with pathological findings, we compared the age of patients with and without each pathological outcome using Wilcoxon rank-sum test, given non-parametric distributions. When the outcome was classified as absent, moderate and severe, we compared the age of these three groups using Kruskal-Wallis test. We also stratified patients by age group: children (≤19y), young adults (20–39y), adults (40-59y) and elderly (≥ 60y). As young adulthood does not have a universal definition, we opted for a wider age range definition, previously used elsewhere (28) in order to increase our sample and to keep a 20-year interval between each age group. We compared frequencies of pathological findings between different age groups using Fisher exact test.

We performed principal component analysis (PCA) and hierarchical clustering on pulmonary and heart pathological variables to try to understand intrinsic patterns in pathological data and summarize them. We then investigated if age correlates independently with the principal components of pathological variables after adjusting for other clinical-demographic variables, by performing three multiple linear regression models with first, second and third principal components (PC1, PC2 and PC3) as the dependent (outcome) variables and clinical-demographic variables as independent (explanatory) variables.

The software *R version 4.1.2* was used for all statistical analysis. All data are available with the corresponding author based on reasonable requests.

## 3 Results

### 3.1 Demographic and clinical characteristics

Eighy-six patients were included in this study, with a median age of 55 years (32.3–66.0). Among them, there were 7 (8.1%) children (≤19y), 19 (22.1%) young adults (20–39y), 22 (25.6%) adults (40-59y) and 38 (44.2%) elderly (≥ 60y). Forty-three (50.0%) patients were women; four (4.7%) of them were pregnant on admission and had deliveries during hospitalization. They had a median body mass index of 25.9 (22.7–29.4) and had a median disease duration of 22 (12–31) days. The most frequent comorbidities were hypertension (40; 46.5%) and diabetes mellitus (23; 26.7%). These comorbidities were more prevalent in older patients; the youngest patient with hypertension was 27 years-old and the youngest diabetic patient was 33 years-old. Twenty-one (24.4%) patients had chronic heart disease, predominantly of hypertensive and ischemic etiology; one child and one young adult had congenital heart disease; 11 (12.8%) patients had previous vascular disease including cerebrovascular disease, peripheral artery disease or thromboembolic events. Among the seven children, four were previously healthy, one had Edwards Syndrome with patent foramen ovale and muscular ventricular septal defects, one had adrenal carcinoma, and one patient had juvenile dermatomyositis on steroids and immunosuppressive medication.

Most patients were hospitalized due to respiratory failure and died of severe COVID-19 pneumonia, refractory to mechanical ventilation, and with multiple organ failure. Four (4.4%) children met criteria for MIS-C and two (2.3%) young adults met criteria for MIS-A. All patients received intensive care. Table 1 shows the demographic, clinical and pathological data.

**Table 1:** Demographic, clinical and pathological characteristics of patients that died of COVID-19 and were submitted to autopsy.

Demographic and clinical characteristics	Children (*n* = 7)	Young Adults (*n* = 19)	Adults (*n* = 22)	Elderly (*n* = 38)	Total (*n* = 86)
Age (y)	11 (8.0–15.5)	32 (22–35)	55.5 (46–54.8)	67.5 (64–74)	55 (32.25–66.0)
Female sex	6 (85.7%)	12 (63.2%)	9 (40.9%)	16 (42.1%)	43 (50.0%)
Puerperal women	0	4 (21.1%)	0	0	4 (4.7%)
Body mass index (kg/m2)	21.4 (17.6–24.9)	26 (24.7–29.4)	28.0 (23.9–29.6)	24.2 (22.7–28.2)	25.9 (22.7–29.4)
Interval symptoms-to-death (days)	10 (6–18)	24.5 (16.3–45.0)	24 (16–28.8)	19 (11.3–31.5)	22 (12–31)
Hypertension	0	2 (10.5%)	11 (50.0%)	27 (71.1%)	40 (46.5%)
Diabetes	0	2 (10.5%)	6 (27.3%)	15 (39.5%)	23 (26.7%)
Smoking	0	3 (15.8%)	4 (18.2%)	14 (36.8%)	21 (24.4%)
Chronic Heart Disease	1 (14.3%)	3 (15.8%)	4 (18.2%)	13 (34.2%)	21 (24.4%)
Immunosuppression	2 (28.6%)	5 (26.3%)	8 (36.4%)	1 (2.6%)	16 (18.6%)
Previous vascular diseases	1 (14.3%)	2 (10.5%)	2 (9.1%)	6 (15.8%)	11 (12.8%)
Neoplasia	1 (14.3%)	2 (10.5%)	4 (18.2%)	3 (7.9%)	10 (11.6%)
Chronic kidney disease	0	1 (5.3%)	4 (18.2%)	1 (2.6%)	6 (7.0%)
Chronic obstructive pulmonary disease	0	0	1 (4.5%)	4 (10.5%)	5 (5.8%)

Pathological findings	Children	Young adults	Adults	Elderly	Total
*Lungs*	*n* = 7	*n* = 19	*n* = 22	*n* = 37	*n* = 85
Acute diffuse alveolar damage					
Severe	4 (57.1%)	7 (36.8%)	11 (50.0%)	23 (62.2%)	45 (45.9%)
Moderate	1 (14.3%)	12 (63.2%)	11 (50.0%)	14 (37.8%)	38 (44.7%)
None	2 (28.6%)	0	0	0	2 (2.4%)
Proliferative diffuse alveolar damage					
Severe	1 (14.3%)	6 (31.5%)	11 (50.0%)	21 (56.8%)	39 (45.9%)
Moderate	0	6 (31.5%)	8 (36.4%)	13 (35.1%)	27 (31.8%)
None	6 (85.7%)	7 (36.8%)	3 (13.6%)	3 (8.1%)	19 (22.4%)
Squamous metaplasia					
Severe	1 (14.3%)	1 (5.3%)	5 (22.7%)	11 (29.7%)	18 (21.2%)
Moderate	0	2 (10.5%)	1 (4.5%)	8 (21.6%)	11 (12.9%)
None	6 (85.7%)	16 (84.2%)	16 (72.7%)	18 (48.6%)	56 (65.9%)
Viral atypia					
Severe	3 (42.9%)	9 (47.4%)	17 (77.3%)	28 (75.7%)	57 (67.1%)
Moderate	4 (57.1%)	9 (47.4%)	5 (22.7%)	9 (24.3%)	27 (31.8%)
None	0	1 (5.3%)	0	0	1 (1.2%)
Vascular thrombosis					
Severe	3 (42.9%)	8 (42.1%)	8 (36.4%)	14 (37.8%)	33 (38.8%)
Moderate	1 (14.3%)	3 (15.8%)	4 (18.2%)	12 (32.4%)	20 (23.5%)
None	3 (42.9%)	8 (42.1%)	10 (45.5%)	11 (29.7%)	32 (37.6%)
“Cystic” formation	1 (14.3%)	2 (10.5%)	4 (18.2%)	6 (16.2%)	13 (15.5%)
Angiomatoid pattern	2 (28.6%)	9 (47.4%)	2 (9.1%)	1 (2.7%)	14 (16.7%)
*Heart*	*n* = 7	*n* = 18	*n* = 22	*n* = 37	*n* = 84
Acute ischemia	4 (57.1%)	10 (52.6%)	7 (31.8%)	6 (16.2%)	27 (32.1%)
Myocarditis	4 (57.1%)	4 (21.1%)	4 (18.2%)	5 (13.5%)	17 (20.2%)
Thrombus	1 (14.3%)	4 (21.1%)	1 (4.5%)	3 (8.1%)	9 (10.7%)

### 3.2 Association between age and pathological findings

**Figure 1:**
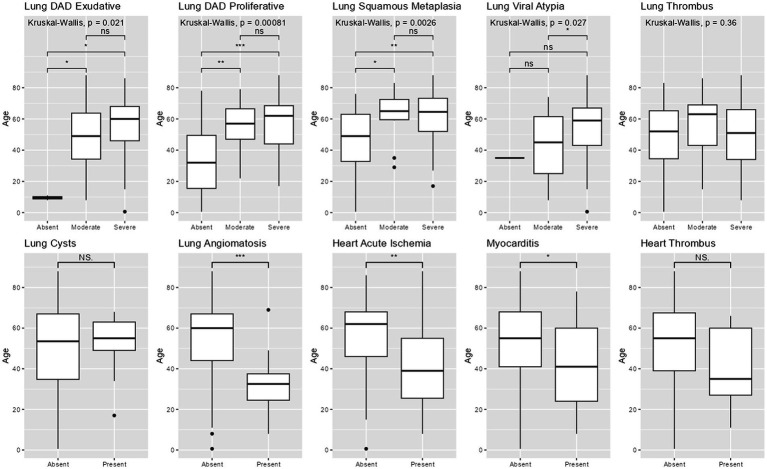
Pathological findings by age. Legend: Boxplots comparing age between groups with different pathological findings. 3-group comparisons used Kruskal-Wallis. 2-group comparisons used Wilcoxon-sum rank. DAD = diffuse alveolar damage. ^***^ = *p* < 0.001; ^**^ = *p* < 0.01; ^*^ = *p* < 0.05; NS = non-significant.

Age was significantly lower in patients with acute heart ischemia (*p* = 0.004), myocarditis (*p* = 0.03) and lung angiomatosis (*p* < 0.001), compared to patients without those findings. Age was significantly higher in patients with alveolar squamous metaplasia (*p* = 0.003), exudative diffuse alveolar damage (*p* = 0.02), proliferative diffuse alveolar damage (*p* < 0.001) and severe viral atypia (*p* = 0.03) (Figure 1).

**Figure 2:**
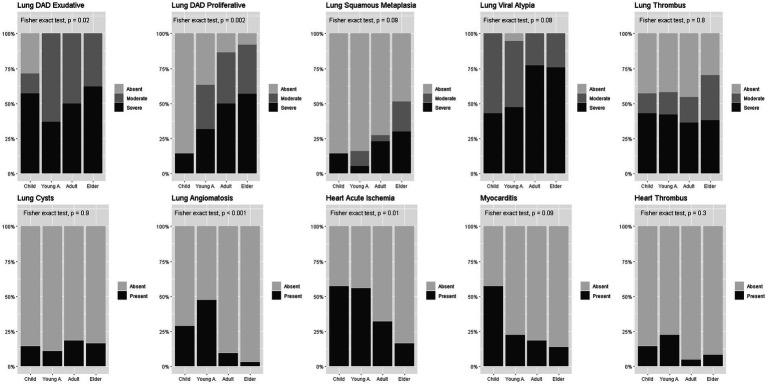
Pathological findings prevalence in age-groups. Legend: Percent stacked bar plots comparing proportion of pathological findings between age groups. Fisher exact test used for comparisons. DAD = diffuse alveolar damage.

Figure 2 shows the frequency of pathological findings in the different age groups. Cardiovascular alterations were more prevalent in younger groups than in older groups, while pulmonary findings were more prevalent and more severe in older groups than in younger ones. Despite the differences, in all groups pulmonary findings were the predominant ones.

**Figure 3:**
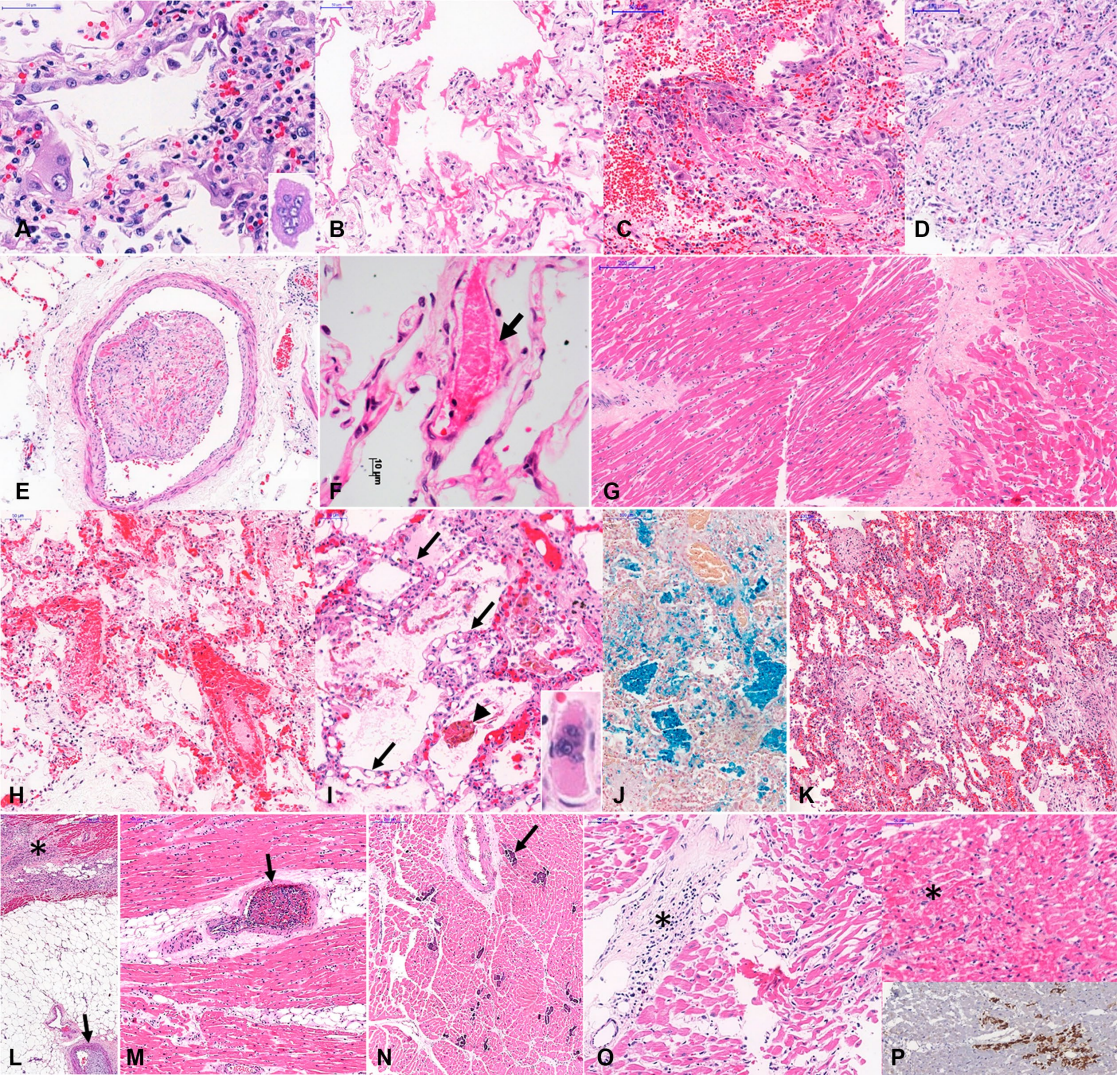
Histopathological findings in COVID-19 related deaths. Legend: Histopathological findings in COVID-19 related deaths, in different age groups. *A to G. Typical COVID-19 autopsy findings in elder patients*: **(A)**-Type II pneumocyte with hyperplasia, atypia and multinuclear cells (inset) and interstitial mononuclear pneumonia; **(B)**-Exudative diffuse alveolar damage; **(C)**-mixed diffuse alveolar damage; **(D)**-Fibroproliferative diffuse alveolar damage; **(E)**-Organized pulmonary thrombus; **(F)**-Earlier fibrin thrombus within pulmonary interalveolar septal capillary, and partial necrosis of endothelial layer (arrow); **(G)**-Myocardial fibrosis and cardiomyocyte hypertrophy. **(H–P)** *Some particular autopsy findings in young patients, with fatal COVID-19:* **(H)**- Earlier fibrin thrombi within pulmonary septal vessels, intense congestion, alveolar edema and little intra-alveolar fibrin exudation in a child with myocarditis and only focal pulmonary lesion; **(I)**-Angiomatoid pattern observed in interalveolar septa associated with fibrinous microthrombi within septal capillaries (arrows) and alveolar edema in a young adult with cardiac failure. **(J)**- Intra-alveolar cluster of hemosiderin-laden alveolar macrophages, stained in blue; **(K–N)**: A 32 year old man without co-morbidities developed severe COVID-19 pneumonia, associated with bronchiolitis obliterans with organizing pneumonia **(K)**. This patient evolved to severe cardiac dysfunction, and the heart samples showed subacute subepicardial infarction (**L**, asterisks) associated with coronary thrombosis (**L**, arrow), intramyocardial arteries with thrombosis (**O**, arrow), and foci of necrotic cardiomyocytes with dystrophic calcification (**N**, arrow); **(O,P)**: A 11 year old girl with fatal myocarditis (**O**, asterisks), with foci of cardiomyocytes with hyaline necrosis (**P**, asterisk on upper panel), stained by anti-C4d complement component (**P**, bottom panel). Staining: **A-I**, **K-O**: hematoxylin–eosin; J: Perls-Prussian blue; P: immunohistochemistry for C4d. Magnification: A,F: 400X; B,C,D,G,H,I,O: 200X, E,J,K,L,M,N,P: 100X.

Figure 3 shows representative images of the main histopathological findings in the different age groups.

### 3.3 Multivariable analysis

**Figure 4:**
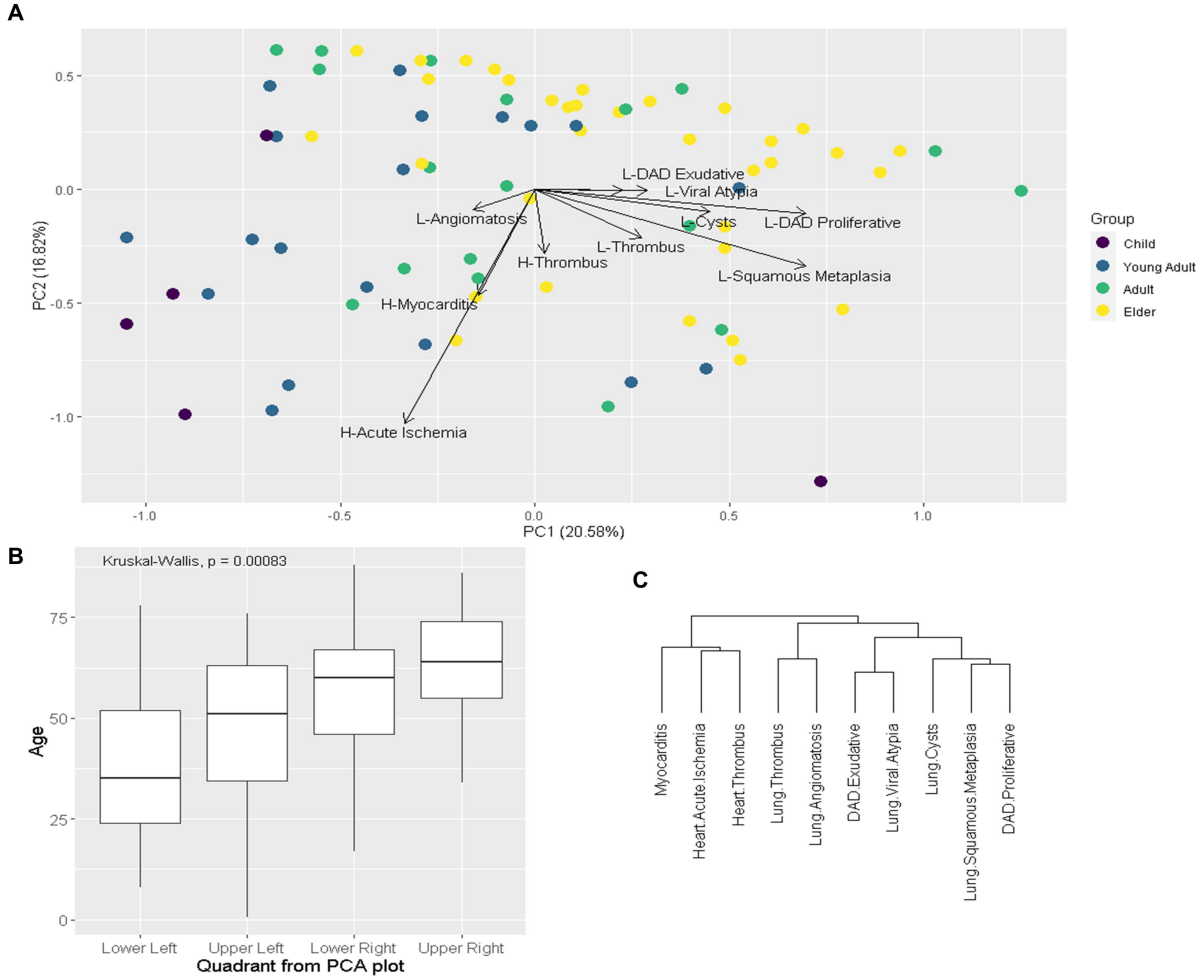
Exploratory multivariate analysis of pathological variables. Legend: **(A)**: Principal component analysis (PCA) of pathological variables, eigenvectors represent the variables, patients stratified by age group. Child: ≤19y; Young Adult: 20–39y; Adult 40-59y; Elder ≥60y. **(B)**: Boxplot comparing age between PCA (A) quadrants. **(C)**: hierarchical cluster of variables. H: Heart; L: Lung; DAD: diffuse alveolar damage.

PCA and hierarchical cluster analysis of pathological variables are presented in Figure 4. PC1 was driven by higher prevalence of most pulmonary findings and lower prevalence of cardiovascular findings, while PC2 was driven by lower prevalence of most cardiovascular findings. We stratified patients by age group in PCA plot, showing that younger age groups tend to concentrate on the lower-left quadrant (lower PC1 and PC2), which is the one with the lowest median age. Hierarchical cluster analysis of pathological variables showed that cardiovascular findings tend to cluster together while pulmonary ones do the same.

We then evaluated if age independently correlates with pathological findings after adjusting for other clinical-demographic variables. We extracted from PCA the first three PCs (PC1, PC2 and PC3), which are variables that summarize the pathological findings, and performed multivariable linear regression models using PCs as dependent (outcome) variable and clinical-demographic characteristics as independent (explanatory) variables. Age was the only explanatory variable that independently correlated with PC1. Neoplasia had a borderline correlation with PC2 and pregnancy independently correlated with PC3. Linear models are presented in Table 2.

**Table 2:** Linear multivariable models assessing the association of clinical and demographic characteristics with principal components of pathological outcomes.

	PC1			PC2			PC3		
Variable	β	SE	*p*-value	β	SE	*p*-value	β	SE	*p*-value
Age	0.02	0.004	0.00002	−0.005	0.003	0.12	0.001	0.003	0.72
Male sex	0.04	0.129	0.74	0.05	0.126	0.72	−0.02	0.117	0.88
BMI	0.005	0.010	0.60	−0.004	0.010	0.70	−0.007	0.009	0.44
Hypertension	−0.28	0.143	0.054	0.03	0.139	0.82	0.15	0.129	0.25
Pregnancy	−0.09	0.277	0.75	−0.12	0.270	0.64	−0.92	0.251	0.0005
Diabetes	−0.10	0.167	0.55	0.11	0.163	0.50	−0.05	0.151	0.76
Chronic heart disease	0.15	0.179	0.41	0.02	0.174	0.93	−0.26	0.162	0.11
Smoking	−0.17	0.161	0.29	0.10	0.157	0.54	0.12	0.146	0.43
Previous vascular diseases	−0.34	0.193	0.08	−0.02	0.188	0.90	0.02	0.175	0.92
Immunosuppression	−0.07	0.175	0.70	0.27	0.170	0.11	0.14	0.158	0.37
COPD	0.20	0.277	0.48	0.27	0.269	0.32	−0.06	0.250	0.80
CKD	0.20	0.268	0.46	−0.20	0.260	0.45	−0.08	0.242	0.75
Neoplasia	0.08	0.184	0.68	0.36	0.179	0.049	−0.12	0.167	0.46
Interval symptoms-to-death	0.0004	0.002	0.86	0.0002	0.002	0.94	−0.002	0.002	0.42

β-Regression coefficient. SE – standard error. PC1, PC2, PC3 – Principal components 1,2 and 3 extracted from Principal Component Analysis. BMI – body mass index. CKD - Chronic kidney disease. COPD - Chronic obstructive pulmonary disease.

## 4 Discussion

We showed that age influences pathological findings in fatal cases of COVID-19. Younger age is mostly associated with cardiovascular findings while older age is associated with pulmonary changes. This is the first COVID-19 autopsy study to assess the whole age spectrum, including young adults and children.

Age has a big impact in a large set of physiological processes, including immune response to infections, and literature on its impact on COVID-19 clinical outcomes is extensive (2, 3). Therefore, we hypothesized that patient’s age would influence pathological outcomes in fatal cases. We showed in bivariate analysis that age is associated with multiple individual pathological findings. After stratifying by age groups, we could also see a significant difference in the frequency of multiple pathological abnormalities. Regression models showed that age independently correlates with such outcomes (summarized in PCs), even after adjusting for other clinical and demographic characteristics. These findings show that age, besides being an important prognostic factor in the severity of COVID-19, also contributes to determining the pathological phenotype.

There are differences in immunopathological mechanisms between children and adults, including: lower ACE2 expression in nasal epithelium, lower ACE2 to SARS-COV-2 affinity and lower TPMRSS2 expression in children; more efficient innate immunity, due to *trained immunity* and better interferon type I response in children; stronger adaptive T-cell response in children, with less frequent lymphopenia (8, 29–31). Most of these explain less severe cases in children, but do not fully explain differences in pathological picture among severe cases. There is data showing that even when considering severe cases, children show higher serum levels of IL17-A and IFNγ (32). Resident memory cells producing these two cytokines have been described in the lungs and a protective role for T helper 17 cells has been described in pulmonary infections, which could help explain the lower rate of pulmonary findings in younger patients (32–34). The greater prevalence of comorbidities and reduced physiological reserve in older patients may also contribute to explain such results. Children also had a shorter duration of disease; however, the symptoms-death interval was not associated with pathological outcomes in the multivariable analysis.

Cardiovascular abnormalities are frequently reported in COVID-19 and associated with worse prognosis (35–37). Clinically, they can manifest as heart failure, arrhythmias, shock and acute coronary syndromes. Both direct viral effects and indirect mechanisms are implicated in acute myocardial injury, assessed clinically through troponin levels and heart imaging, and histologically, through autopsy (38). Myocarditis and acute ischemia are among the most relevant findings that can be assessed through pathological examination (39). In our cohort, we found myocarditis in 20.2% of the patients and acute ischemia in 32.1%. As MIA-US does not allow complete organ examination, it is possible that these frequencies were underestimated. There is uncertainty on the clinical relevance of those pathological findings, since they were not always clinically diagnosed. Most patients experienced severe respiratory distress and shock before death, and it is difficult to predict the contribution of cardiovascular abnormalities in the entire clinical picture. Yet, we know from previous studies that cardiovascular injury is a prognostic marker in COVID-19 (36, 37). Both myocarditis and acute ischemia share inflammatory mechanisms. COVID-19-associated myocyte necrosis is mainly caused by microthrombi with greater fibrin and terminal complement C5b-9 immunostaining than non-COVID cases (26). These findings were more prevalent in children and young adults in our study. A metanalysis showed that cardiovascular findings are common in pediatric autopsies, and suggested they were more prevalent than adult studies (40). It is likely that COVID-19 fatal cases in younger patients are related to highly unregulated immune responses, as seen in MIS-C, which is a rare complication of COVID-19 with a still poor understood pathophysiology (41, 42). Few cases of MIS-A have also been reported (43–45). These inflammatory syndromes, that are part of the severe COVID-19 spectrum, occur more frequently in children and are associated with extrapulmonary damage, such as cardiovascular complications, which likely contributed to our results (15, 16). However, MIS-C and MIS-A are probably not the only explanation for our findings, as we reported an increased prevalence of cardiovascular damage in the young adult group, among which only two MIS-A were described.

We did not find differences between age groups on the prevalence of thrombosis, neither in the heart nor in the lungs. The occurrence of thrombotic complications is one of the hallmarks of COVID-19, with severely ill patients frequently showing abnormal coagulation tests and thrombotic disease, especially in the lungs (46–48). Early in the pandemic, we have described the presence of lung microthrombi in a large proportion of autopsies of individuals with fatal COVID-19 (49). A systematic review has shown that clinical thrombosis is rarer in children compared to adults, but still requires clinical surveillance, especially in the lungs (50). In the present series, we found a high prevalence (63.4%) of lung thrombosis and a lower rate (10.7%) in hearts. This prevalence may be underestimated because of the MIA-US protocol. Pro-thrombotic mechanisms in the lungs are diverse and include both systemic and local processes (48). Our results suggest that in very severe cases, these processes are active irrespectively of age.

Our findings may impact patient care. Clinicians facing severe cases of COVID-19 in children or young adults should assess their cardiac function. In many cases, hemodynamic instability in critical patients with COVID-19 is empirically attributed to systemic inflammation related to the pulmonary disease, while careful examination of other causes of shock, such as cardiogenic, is not performed. This assessment could lead to better supportive measures and other therapeutic approaches.

This study has some limitations. Laboratory data was not available in many cases, especially from the hospital admission of patients transferred from lower-complexity hospitals after clinical deterioration. This precludes laboratory-pathological correlation. Minimally invasive autopsy has limitations regarding tissue representation in comparison to conventional autopsy. Tissue samples are collected through needle punctures, guided by ultrasound analysis, but without direct macroscopic examination. This may lead to decreased diagnostic sensitivity for pathological findings that are not diffuse throughout organs, especially when considering the heart, underestimating the prevalence of some findings. To mitigate this issue, we performed multiple punctures in each organ and followed a standardized protocol. The small number of children compared to other age-groups may limit the extrapolation of some findings. We must consider that most children with COVID-19 have a mild or asymptomatic disease; therefore, the different pathological presentations reported in this study refer only to severe/fatal cases.

In conclusion, we showed that age modulates pathological findings in patients with fatal COVID-19. Younger patients showed more cardiovascular abnormalities and less pulmonary changes at autopsy compared to older patients. Further research is needed to explore the mechanisms that could explain these findings. Our results may impact younger patients’ care in severe cases by encouraging physicians to assess more carefully the cardiac function of these patients.

## Data availability statement

The raw data supporting the conclusions of this article will be made available by the authors, without undue reservation.

## Ethics statement

The studies involving humans were approved by Comissão de Ética para Análise de Projetos de Pesquisa do HCFMUSP, affiliated with Hospital das Clinicas, University of São Paulo Medical School (protocol number 3.951.904). The studies were conducted in accordance with the local legislation and institutional requirements. Written informed consent for participation in this study was provided by the participants' legal guardians/next of kin.

## Author contributions

FG: Formal analysis, Methodology, Writing – original draft. AD-N: Formal analysis, Methodology, Writing – original draft. LS: Investigation, Methodology, Writing – review & editing. RM: Investigation, Writing – review & editing. TM: Investigation, Methodology, Writing – review & editing. PS: Funding acquisition, Investigation, Methodology, Writing – review & editing. MD: Conceptualization, Funding acquisition, Investigation, Methodology, Supervision, Writing – original draft.

## Footnotes

^1^ https://pisa.hc.fm.usp.br/
